# Liquid-State Interfacial Reactions of Lead-Free Solders with FeCoNiCr and FeCoNiMn Medium-Entropy Alloys at 250 °C

**DOI:** 10.3390/ma18102379

**Published:** 2025-05-20

**Authors:** Chao-Hong Wang, Yue-Han Li

**Affiliations:** Department of Chemical Engineering, National Chung Cheng University, Chiayi 621301, Taiwan

**Keywords:** medium-entropy alloys, high-entropy alloys, lead-free solders, interfacial reaction, wettability

## Abstract

This study investigates the interfacial reactions of FeCoNiCr and FeCoNiMn medium-entropy alloys (MEAs) with Sn and Sn-3Ag-0.5Cu (SAC305) solders at 250 °C. The evolution of interfacial microstructures is analyzed over various aging periods. For comparison, the FeCoNiCrMn high-entropy alloy (HEA) is also examined. In the Sn/FeCoNiCr system, a faceted (Fe,Cr,Co)Sn_2_ layer initially forms at the interface. Upon aging, the significant spalling of large (Fe,Cr,Co)Sn_2_ particulates into the solder matrix occurs. Additionally, an extremely large, plate-like (Co,Ni)Sn_4_ phase forms at a later stage. In contrast, the Sn/FeCoNiMn reaction produces a finer-grained (Fe,Co,Mn)Sn_2_ phase dispersed in the solder, accompanied by the formation of the large (Co,Ni)Sn_4_ phase. This observation suggests that Mn promotes the formation of finer intermetallic compounds (IMCs), while Cr facilitates the spalling of larger IMC particulates. The Sn/FeCoNiCrMn system exhibits stable interfacial behavior, with the (Fe,Cr,Co)Sn_2_ layer showing no significant changes over time. The interfacial behavior and microstructure are primarily governed by the dissolution of the constituent elements and composition ratio of the HEAs, as well as their interactions with Sn. Similar trends are observed in the SAC305 solder reactions, where a larger amount of fine (Fe,Co,Cu)Sn_2_ particles spall from the interface. This behavior is likely attributed to Cu doping, which enhances nucleation and stabilizes the IMC phases, promoting the formation of finer particles. The wettability of SAC305 solder on MEA/HEA substrates was further evaluated by contact angle measurements. These findings suggest that the presence of Mn in the substrate enhances the wettability of the solder.

## 1. Introduction

High-entropy alloys (HEAs), composed of five or more principal elements in near-equiatomic ratios, have attracted widespread attention due to their unique combination of high strength and toughness [1,2,3]. Unlike conventional alloys, which are typically based on one or two major elements, HEAs are stabilized by high mixing entropy, lowering the Gibbs free energy and favoring the formation of simple solid solution phases. In addition to their excellent mechanical properties, HEAs also demonstrate superior thermal stability and corrosion resistance, making them highly suitable for use in harsh environments such as aerospace, nuclear power systems, and marine engineering [4,5]. Among the various HEA systems, FeCoNiCrMn has emerged as one of the most extensively studied, featuring a stable FCC structure [6]. Furthermore, refractory HEAs such as TiNbZrMoV and MoNbTaVW primarily form a single BCC solid solution and exhibit excellent high-temperature strength and phase stability [7,8].

Compared to high-entropy alloys, medium-entropy alloys exhibit lower mixing entropy, typically ranging from *R* to 1.5*R* (where *R* is the gas constant). MEAs generally comprise three to four principal elements in near-equiatomic proportions, representing an intermediate class of multi-component alloys that bridge the gap between conventional alloys and HEAs [9,10]. Unlike HEAs, MEAs possess relatively lower compositional complexity, which allows for better control over microstructure and phase stability while still retaining desirable mechanical and thermal properties. Among these, CoCrNi-based MEAs have attracted considerable attention in recent years, driven by their promising performance and the rapid increase in related research efforts [11,12,13]. They exhibit not only excellent mechanical strength and ductility at elevated temperatures but also outstanding fracture toughness at cryogenic temperatures [13].

With the miniaturization of solder joints, the reliability of micro-solder joints in electronic devices has become an increasing concern. Ni is commonly used as a diffusion barrier material to prevent excessive reactions between the solder and Cu pads, ensuring better long-term performance of the joint [14,15]. In addition, lead-free solders, which are environmentally friendly, particularly Sn–Ag and Sn–Ag–Cu alloys, have gained widespread use over the past two decades. Recent studies [16,17,18,19,20,21] have highlighted the potential of HEAs as next-generation diffusion barrier materials in microelectronic and semiconductor applications, owing to their inherently sluggish diffusion characteristics. The low atomic mobility in HEAs effectively suppresses interfacial reactions, limits undesirable interdiffusion, and resists electromigration-induced degradation when in contact with other materials, such as solder [22,23,24,25,26]. As a result, the growth of IMCs is significantly inhibited, enhancing the long-term reliability and overall performance of solder joints. Furthermore, HEAs and MEAs have emerged as promising structural materials for demanding industrial applications, such as turbine blades and critical components in marine and aerospace engineering. Since joining or bonding processes are inevitable in many of these applications, increasing attention has been directed toward studies of the associated interfacial reactions [27,28,29,30,31].

A previous study [30] on the FeCoNiCrMn HEA demonstrated that, when reacted with lead-free solder systems, the interfacial (Fe,Cr,Co)Sn_2_ phase exhibits excellent microstructural and thermal stability. This phase grows slowly and does not exhibit IMC spalling during prolonged soldering, even at elevated temperatures up to 500 °C. Such stable interfacial behavior highlights the potential of FeCoNiCrMn HEA as an effective diffusion barrier layer in soldering applications. The presence of Cr and Co in the (Fe,Cr,Co)Sn_2_ phase indicates the significant solubility of these elements in the FeSn_2_ structure, likely due to substitutional incorporation into Fe lattice sites. Furthermore, the medium-entropy alloy FeCoNiMn, when soldered with Sn–3.0Ag–0.5Cu (SAC305), has shown favorable wettability characteristics [31], further supporting the potential of FeCoNiMn MEA as a reliable candidate for microelectronic interconnect applications. Accordingly, the complex composition of HEAs, with their multiple constituent elements, can significantly influence their dissolution behavior and the subsequent formation of IMCs during the soldering process.

Despite these promising findings, studies on the interfacial reactions between the FeCoNiCrMn alloy family and lead-free solders remain limited. Further research is needed to elucidate the underlying interaction mechanisms and the specific roles of individual elements in HEAs during the soldering process. This study presents a comparative analysis of FeCoNiCr and FeCoNiMn MEAs in their interactions with pure Sn and SAC305 lead-free solders at 250 °C. By systematically examining the interfacial microstructures and the evolution of IMCs over different aging periods, the research aims to investigate the contributions of Cr and Mn to interfacial stability and IMC formation. Additionally, the FeCoNiCrMn HEA is included as a reference to benchmark the performance of the FeCoNiCr and FeCoNiMn MEAs. The findings are expected to offer valuable insights for the design of HEAs optimized for enhanced solder joint reliability in advanced electronic applications.

## 2. Materials and Methods

FeCoNiCr, FeCoNiMn, and FeCoNiCrMn (MEA/HEA) alloys were prepared using high-purity elements (99.99 wt. %). The constituent elements were precisely weighed according to the desired compositions, with a weighing error of ±0.001 g. Each alloy was prepared with a total weight of 5.000 ± 0.004 g. The alloy was subsequently melted using an arc-melting furnace under an argon atmosphere. To ensure the compositional homogeneity, the ingot was flipped and re-melted at least five times. The SAC305 solder was also prepared from high-purity constituent elements with a total weight of 5.000 ± 0.003 g. The solder alloy was sealed in a quartz tube under a vacuum of 10^−2^ torr, homogenized in a furnace at 800 °C for 3 days, and subsequently quenched in water. For the subsequent interfacial reaction experiments, the Sn or SAC solder alloy were cut into 2 mm-thick pieces using a low-speed precision cutter, while the MEA/HEA substrates were sectioned into 1 mm-thick slices. These alloy discs were ground and polished to ensure a smooth and clean surface.

Before conducting the reactions, the MEA/HEA substrates were cleaned with rosin-based flux. A solder piece was then placed on the MEA/HEA substrate, and heated on a hot plate at 250 ± 1 °C for several seconds to perform the pre-assembly procedure. The interfacial reactions were performed at 250 °C for different durations. Each reaction time point was obtained from a separate specimen. To further confirm the reproducibility of the results, additional specimens were prepared and analyzed under identical experimental conditions, consistently exhibiting a similar microstructure. The samples were encapsulated in epoxy resin, followed by grinding and polishing for metallographic observation. To better examine the interfacial microstructure, all reaction couples were gently etched to remove the solder using a Sn-etching solution. The interfacial microstructure was examined using scanning electron microscopy (SEM) in back-scattered electron image (BEI) mode. The compositional analysis of the reaction phases was conducted using an electron probe microanalyzer (EPMA, JEOL JXA-8200, Tokyo, Japan). Additionally, X-ray diffraction (XRD, Bruker D8, Billerica, MA, USA) with Cu–K_α_ radiation (λ = 1.54056 Å) was utilized to identify and analyze the reaction phases. The solder in the reaction couples was entirely removed through deep etching with a Sn-etching solution, thereby revealing the underlying IMCs. The resulting X-ray diffraction patterns were identified by comparison with the reference data from the Joint Committee on Powder Diffraction Standards (JCPDS) database (PCPDFWIN version 2.3).

The wettability between SAC305 solder and the MEA/HEA substrates was evaluated by contact angle measurements. FeCoNiCr, FeCoNiMn, and FeCoNiCrMn substrates (5 × 5 × 1 mm^3^) were ultrasonically cleaned in ethanol and dried with nitrogen gas. A small SAC305 solder ball (0.5 mm in diameter) was placed on each substrate, and the assemblies were heated in a quartz tube furnace under a flowing argon (Ar) atmosphere at 250 ± 1 °C for 20 s to achieve thermal equilibrium. After heating, the samples were slowly cooled to room temperature under the same inert atmosphere to minimize oxidation. The solder joints were then sectioned at the midpoint and polished to expose the interface between the solder and substrate. The contact angle was measured using an optical microscope, with three replicate measurements taken for each substrate to ensure reproducibility.

## 3. Results and Discussion

### 3.1. Sn/FeCoNiCr Reactions

**Figure 1:**
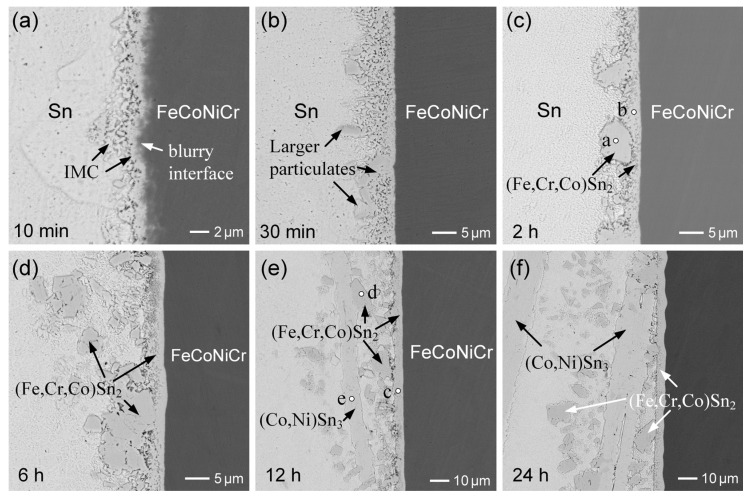
BEI micrographs of interfacial microstructures in Sn/FeCoNiCr reactions at 250 °C for (**a**) 10 min, (**b**) 30 min, (**c**) 2 h, (**d**) 6 h, (**e**) 12 h, and (**f**) 24 h. The points labeled a–e represent the analyzed positions for EPMA, with the results summarized in Table S1.

Figure 1a–f present the BEI micrographs illustrating the interfacial microstructural evolution in the Sn/FeCoNiCr reactions at 250 °C over aging durations ranging from 10 min to 24 h. After 10 min of reaction, as shown in Figure 1a, a reaction layer approximately 1.8 ± 0.4 µm thick forms at the interface, exhibiting a loose and porous microstructure. Notably, in the BEI imaging mode, the boundary between the reaction layer and the FeCoNiCr substrate appears blurry and irregular, rather than forming a well-defined, planar interface. This phenomenon is attributed to the non-uniform dissolution rates of the constituent elements in the FeCoNiCr MEA substrate. During the initial stage, the dissolution of the substrate into the molten solder is relatively more pronounced.

Figure 1b shows that after 30 min of aging, the reaction phase grew significantly thicker while retaining a porous and non-dense microstructure. This suggests that the reaction phase consisted of numerous grains, with the pore regions filled with solder. Notably, some larger grains were observed in the reaction phase near the solder. A similar phenomenon was observed in the 2 h sample, as shown in Figure 1c, where the reaction phase particulates, approximately 10 µm in size, appeared to detach from the interface and disperse into the solder. EPMA analysis revealed that the reaction phase (point a) had a composition of 16.5 at.%Fe-9.2 at.%Cr-6.3 at.%Co-1.1 at.%Ni-66.9 at.%Sn. This composition corresponds to the FeSn_2_ phase with high Cr and Co solubilities, and it was labeled as the (Fe,Cr,Co)Sn_2_ phase in this study. The corresponding analysis results are presented in Table S1. The composition of the reaction phase layer (point b) near the FeCoNiCr substrate was 12.7 at.%Fe-13.5 at.%Cr-5.6 at.%Co-1.0 at.%Ni-67.2 at.%Sn, which also corresponds to the (Fe,Cr,Co)Sn_2_ phase.

As the reaction time was extended to 6 h, as shown in Figure 1d, the interfacial (Fe,Cr,Co)Sn_2_ layer with a porous structure exhibited no significant growth, while the severe dispersion of large and dense (Fe,Cr,Co)Sn_2_ particulates was observed. This suggests that the interfacial (Fe,Cr,Co)Sn_2_ layer underwent dissolution, leading to the grain growth of (Fe,Cr,Co)Sn_2_ particulates in the solder. Further prolonging the reaction time to 12 h, as depicted in Figure 1e, revealed more pronounced IMC spalling. Notably, the dispersion phases had two distinct contrasts. The dark phase had a round particulate structure, approximately 10 µm in size, whereas the bright phase had an unusually large plate-like structure, exceeding 100 µm in length. EPMA analysis identified the dark phase as (Fe,Cr,Co)Sn_2_, while the bright phase was confirmed to be (Co,Ni)Sn_3_.

**Figure 2:**
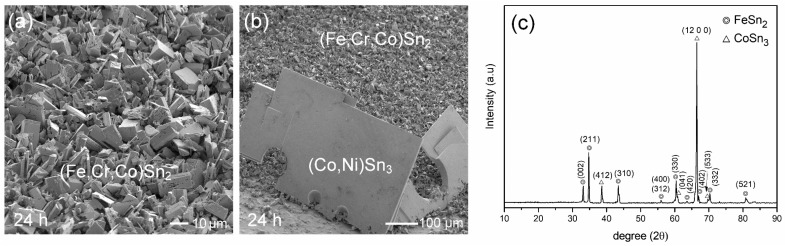
Sn/FeCoNiCr reaction at 250 °C for 24 h after deep-etching. (**a**,**b**) The morphologies of (Fe,Cr,Co)Sn_2_ and (Co,Ni)Sn_3_, respectively. (**c**) The XRD analysis results, indicating the presence of FeSn_2_ and CoSn_3_, as referenced from the JCPDS database (FeSn_2_—#73-2030 and α-CoSn_3_—#48-1813).

The Sn/FeCoNiCr reaction at 250 °C after 24 h exhibited a similar interfacial microstructure. The (Fe,Cr,Co)Sn_2_ phase layer, with a thickness of 6.5 ± 0.4 µm, was present at the interface. Additionally, (Fe,Cr,Co)Sn_2_ particulates and chunky (Co,Ni)Sn_3_ phases were dispersed in the solder matrix near the interface. The series of microstructural features observed at each time point were highly consistent across multiple independent specimens, demonstrating the high reproducibility of the interfacial reaction behavior. Furthermore, the reaction couple was deep-etched to completely remove the solder, exposing the IMC. Figure 2a,b show the morphologies of FeSn_2_ and CoSn_3_, respectively. The (Fe,Cr,Co)Sn_2_ grains displayed a well-faceted polyhedral shape with a diameter of approximately 10 µm, while the (Co,Ni)Sn_3_ appeared as rectangular plate-like structures with sizes on the order of several hundred micrometers.

The XRD analysis presented in Figure 2c shows that most of the distinct diffraction peaks correspond well to FeSn_2_, confirming the presence of the (Fe,Cr,Co)Sn_2_ phase. FeSn_2_ has a body-centered orthorhombic structure (JCPDS #73-2030, S.G. *I4/mcm*, *a* = 0.6520 nm, *c* = 0.5312 nm). The α-CoSn_3_ has an end-centered orthorhombic structure (JCPDS #48-1813, S.G. *Cmca*, *a* = 1.686 nm, *b* = 0.6268 nm, *c* = 0.6270 nm). The XRD spectra of the (Co,Ni)Sn_3_ phase revealed a strong peak at (12, 0, 0), indicating that the normal direction of the plate surface is aligned with the a-axis. These XRD spectra are consistent with the IMC phases observed at the interface.

**Figure 3:**
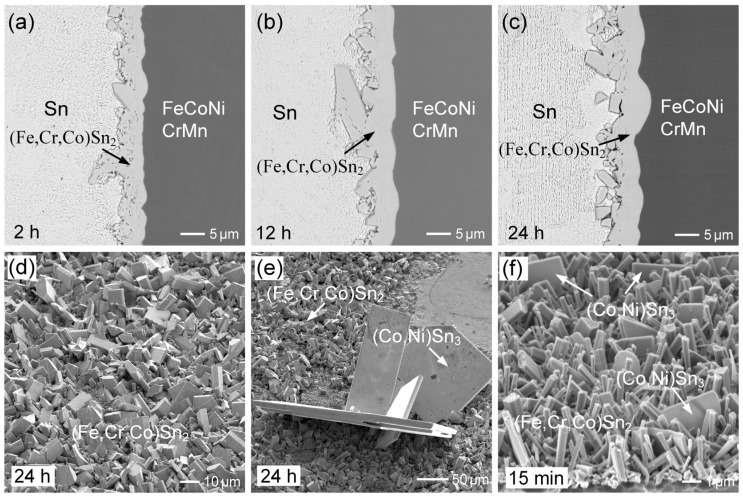
BEI micrographs of interfacial microstructures in Sn/FeCoNiCrMn reactions at 250 °C for (**a**) 2 h, (**b**) 12 h, and (**c**) 24 h. (**d**,**e**) The morphologies of (Fe,Cr,Co)Sn_2_ and (Co,Ni)Sn_3_, respectively, after 24 h of aging. (**f**) The initial morphology of (Fe,Cr,Co)Sn_2_ after 15 min of aging.

The observed results indicate significant IMC spalling in the liquid-state Sn/FeCoNiCr interfacial reactions, suggesting unstable interfacial behavior. In contrast, a prior study [30] on the Sn/FeCoNiCrMn reaction demonstrated the excellent interfacial stability, as no IMC spalling occurred even after prolonged reaction times. To further investigate and clarify the differences in interfacial behavior, the Sn/FeCoNiCrMn reactions were conducted at 250 °C. Figure 3a–c show the interfacial microstructure of the Sn/FeCoNiCrMn reactions at 250 °C after 2 h, 12 h, and 24 h, respectively. As reported in the prior study [30], the dense (Fe,Cr,Co)Sn_2_ reaction phase exhibited no significant growth over the aging period. Notably, the (Fe,Cr,Co)Sn_2_ phase layer remained highly stable without any IMC spalling. These observed interfacial results clearly demonstrate that CoNiCrMn possesses superior interfacial stability compared to FeCoNiCr.

Figure 3d shows the morphology of (Fe,Cr,Co)Sn_2_ in the sample after 24 h of aging. In addition to the faceted columnar morphology exhibited by the (Fe,Cr,Co)Sn_2_ grains, several large (Co,Ni)Sn_3_ grains with plate-like morphologies were also observed. However, the formation of (Co,Ni)Sn_3_ grains was sparse and relatively rare across the entire substrate. The initial formation of (Fe,Cr,Co)Sn_2_, as presented in Figure 3f, also exhibited a faceted pillar-like morphology, with grain diameters of approximately 0.5 µm. Additionally, several thin, plate-like grains were observed, suggested to be (Co,Ni)Sn_3_, with sizes around 3.5 µm. By comparing Figure 2a and Figure 3d, it was found that the grain sizes of (Fe,Cr,Co)Sn_2_ were similar in both systems, suggesting that the presence of Mn in the FeCoNiCrMn alloy did not significantly affect the nucleation and growth behavior of the (Fe,Cr,Co)Sn_2_ phase, compared to the FeCoNiCr reaction.

EPMA analysis indicates that the (Fe,Cr,Co)Sn_2_ phase contained high levels of Cr, moderate amounts of Co, and trace amounts of Ni and Mn. It suggests that most of the Ni and Mn from the substrate, along with a portion of the Co, preferentially dissolved into the solder, whereas Cr remained largely incorporated within the (Fe,Cr,Co)Sn_2_ phase. The differing dissolution behaviors of these transition metals in molten Sn can be reasonably explained by their limiting partial molar enthalpies. The reported values for Fe, Cr, Co, Ni, and Mn in Sn are +34, +32, +1, −13, and −22 (kJ/mol), respectively [32]. The positive values for Fe and Cr indicate repulsive interactions with molten Sn, which is consistent with the Sn–Fe and Sn–Cr phase diagrams, both exhibiting monotectic reactions and limited mutual solubility [33,34]. In contrast, the negative enthalpies for Ni and Mn suggest attractive interactions with Sn, promoting their dissolution. This behavior is also supported by the Sn–Ni and Sn–Mn phase diagrams [35,36]. Co, with an enthalpy value near zero, exhibits an intermediate dissolution behavior in Sn.

In both the FeCoNiCr and FeCoNiCrMn reactions, the dissolution of Co and Ni into molten Sn resulted in supersaturation, which subsequently led to the formation of (Co,Ni)Sn_3_. Interestingly, the dissolved Co and Ni atoms primarily contributed to the growth of a limited number of large, plate-like (Co,Ni)Sn_3_ grains. This observation suggests that the nucleation of (Co,Ni)Sn_3_ is kinetically hindered, likely due to a high nucleation barrier, leading to preferential growth on pre-existing sites rather than the widespread formation of fine grains. Furthermore, the amount of (Co,Ni)Sn_3_ formed in the FeCoNiCr system was significantly greater than that in the FeCoNiCrMn reaction. This difference can be attributed to the higher total concentration of Co and Ni (25 at.%) in FeCoNiCr compared to 20 at.% in FeCoNiCrMn, resulting in a greater availability of these elements for dissolution into Sn and subsequently enhancing the formation of (Co,Ni)Sn_3_.

### 3.2. Sn/FeCoNiMn Reactions

**Figure 4:**
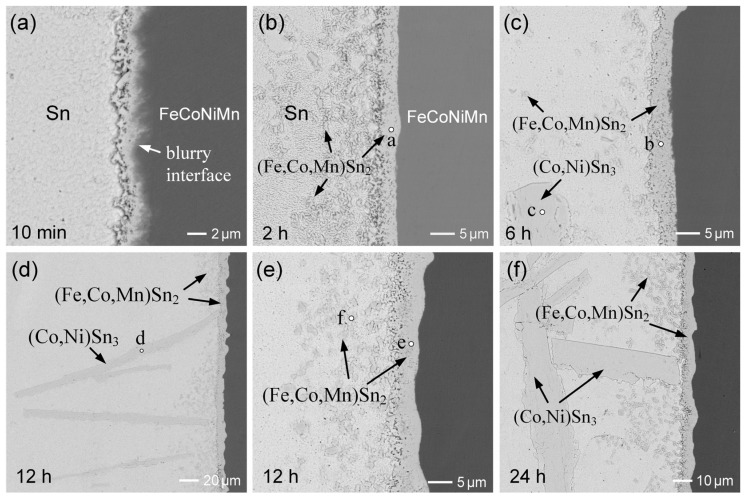
BEI micrographs of interfacial microstructures in Sn/FeCoNiMn reactions at 250 °C for (**a**) 10 min (**b**) 2 h, (**c**) 6 h, (**d**) 12 h (**e**) 12 h (zoom-in image) and (**f**) 24 h. The points labeled a–f represent the analyzed positions for EPMA, with the results summarized in Table S2.

Figure 4a–f present the interfacial microstructures of the Sn/FeCoNiMn reactions aged at 250 °C for various durations. After 10 min of aging, as shown in Figure 4a, a thin interfacial reaction layer with a thickness of 1.9 ± 0.2 µm was formed. Similar to the FeCoNiCr system, the interface between IMC and the FeCoNiMn MEA appeared blurry and irregular, which can be attributed to the non-uniform dissolution of the multiple constituent elements in the MEA. Upon extending the aging time to 2 h (Figure 4b), significant interfacial evolution was observed. The reaction layer developed a porous structure, and numerous fine IMC particulates were dispersed within the solder region adjacent to the interface. At 6 h (Figure 4c), in addition to the porous interfacial IMC layer and fine dispersed phases, a large bright phase emerged in the solder matrix. EPMA analysis revealed that the porous interfacial phase contained 8.7 at.% Co and 5.3 at.% Mn in the FeSn_2_ structure, and it was thus designated as the (Fe,Co,Mn)Sn_2_ phase. The bright chunky phase was identified as (Co,Ni)Sn_3_. The corresponding EPMA data are summarized in Table S2.

**Figure 5:**
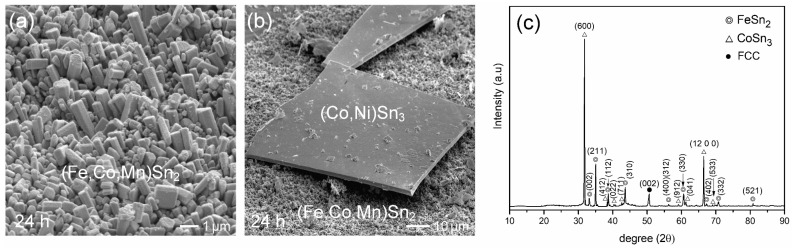
Sn/FeCoNiMn reaction at 250 °C for 24 h after deep-etching. (**a**,**b**) The morphologies of the (Fe,Co,Mn)Sn_2_ and (Co,Ni)Sn_3_, respectively. (**c**) The XRD analysis results, indicating the presence of FeSn_2_ and CoSn_3_, as referenced from the JCPDS database (FeSn_2_—#73-2030 and α-CoSn_3_—#48-1813).

Figure 4d,e display the interfacial microstructures of the Sn/FeCoNiMn reaction after 12 h of aging. Several stripe-shaped (Co,Ni)Sn_3_ phases, approximately 200 µm in length, were observed in the solder matrix, along with numerous fine (Fe,Co,Mn)Sn_2_ particles dispersed near the interface. These fine IMC particulates are presumed to have spalled from the porous (Fe,Co,Mn)Sn_2_ interfacial layer. After 24 h of reaction, a similar interfacial microstructure was maintained, as shown in Figure 4f. The observed microstructural evolutions were highly consistent across all specimens, indicating excellent reproducibility. The consistent features observed across all specimens highlight the excellent reproducibility of the interfacial reaction behavior. Figure 5a,b further present the grain morphologies of the (Fe,Co,Mn)Sn_2_ and (Co,Ni)Sn_3_, respectively. Remarkably, the (Fe,Co,Mn)Sn_2_ grains exhibited a fine faceted pillar-like morphology with diameters below 1 µm, while the (Co,Ni)Sn_3_ phase showed large, plate-like structures. Additionally, the XRD analysis shown in Figure 5c confirms the formation of both Fe (Fe,Co,Mn)Sn_2_ and (Co,Ni)Sn_3_ phases on the FeCoNiMn substrate, consistent with the microstructural observations.

**Figure 6:**
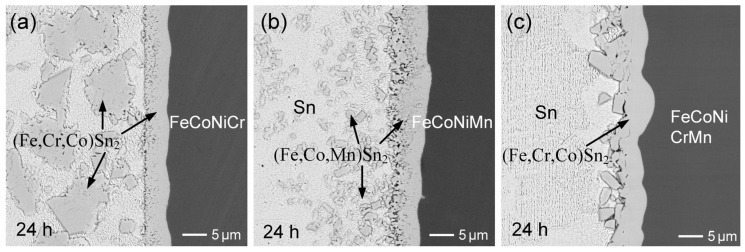
Comparison of the microstructures of the dispersed IMC phases formed during the reactions of Sn with (**a**) FeCoNiCr, (**b**) FeCoNiMn, and (**c**) FeCoNiCrMn substrates. Figure (**c**), identical to Figure 3c, is shown again here for comparison.

In comparison to the reactions involving the three substrates, i.e., FeCoNiCr, FeCoNiMn, and FeCoNiCrMn, the dispersed FeSn_2_ phases exhibited distinct morphologies and behaviors, as illustrated in Figure 6a–c. In the Sn/FeCoNiCrMn system (Figure 6c), the interface remained stable, and no dispersed (Fe,Cr,Co)Sn_2_ particles observed in the solder. In contrast, the Sn/FeCoNiCr system showed the significant dispersion of large (Fe,Co,Cr)Sn_2_; particulates into the solder, while the Sn/FeCoNiMn reaction resulted in only fine, uniformly distributed (Fe,Co,Mn)Sn_2_ particles. In the Sn/FeCoNiCr system, the interfacial (Fe,Cr,Co)Sn_2_ phase layer likely underwent partial dissolution, followed by grain regrowth. Due to the strong repulsive interaction between Cr and molten Sn, the reprecipitated (Fe,Cr,Co)Sn_2_ tends to form larger grains to minimize surface energy. As the reaction progressed, these destabilized IMCs detached from the interface and dispersed into the solder matrix, reflecting the interfacial instability driven by compositional and thermodynamic factors.

In contrast, as shown in Figure 6b, numerous fine (Fe,Co,Mn)Sn_2_ particles were observed dispersed within the solder in the Sn/FeCoNiMn system. This behavior is likely attributed to the attractive interaction between Mn and molten Sn, which facilitates Mn dissolution and may assist in the nucleation and stabilization of FeSn_2_. Consequently, grain growth is suppressed, favoring the formation of finer (Fe,Co,Mn)Sn_2_ particles. The finer morphology also results in a higher surface area-to-volume ratio, which further enhances the thermodynamic favorability of Mn–Sn interactions. On the other hand, the Sn/FeCoNiCrMn reaction exhibited a highly stable interfacial microstructure, with no (Fe,Cr,Co)Sn_2_ particles dispersed in the solder. This suggests a synergistic effect between Cr and Mn on interfacial stability. While Mn promotes elemental dissolution into molten Sn, Cr exhibits a strong repulsive interaction with Sn, thereby suppressing dissolution. As a result, Fe and Cr atoms are predominantly retained in the interfacial IMC layer, effectively preventing spallation and the dispersion of the (Fe,Cr,Co)Sn_2_ phase into the solder. Overall, the distinct interfacial behaviors among the three systems are governed by the dissolution tendencies of the HEA constituent elements and their thermodynamic affinities with Sn. The competitive effects of Cr and Mn play a pivotal role in determining the morphology, distribution, and stability of the interfacial IMCs.

### 3.3. SAC305/FeCoNiCr Reactions

**Figure 7:**
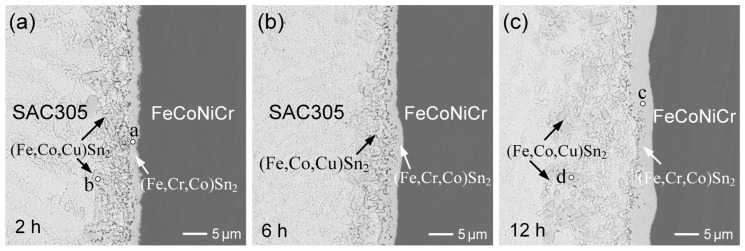
BEI micrographs of interfacial microstructures in SAC305/FeCoNiCr reactions at 250 °C for (**a**) 2 h, (**b**) 6 h, and (**c**) 12 h. The points labeled a–d represent the analyzed positions for EPMA, with the results summarized in Table S3.

The FeCoNiCr MEA substrate was also reacted with SAC305 solder at 250 °C. As displayed in Figure 7a, an irregular IMC phase began to spall into the solder matrix after 2 h of aging. The EPMA analysis results, summarized in Table S3, indicate that the composition of the interfacial region (point a) was 17.4 at.%Fe-10.2 at.%Cr-3.2 at.%Co-66.0 at.%Sn, along with trace amounts of 1.7 at.%Cu and 1.6 at.%Ni, corresponding to the (Fe,Cr,Co)Sn_2_ phase. In contrast, a nearby small dispersed particle (point b) exhibited a composition of 18.2 at.%Fe-7.3 at%Co-4.2 at.%Cu-1.9 at.%Cr-1.8 at.%Ni-66.5 at.%Sn. This IMC was identified as an FeSn_2_-type compound enriched in Co and Cu, with only minor Cr and Ni content, and is thus designated as (Fe,Co,Cu)Sn_2_. Although both IMCs were classified as FeSn_2_-type phases, they exhibited distinctly different chemical compositions. The dispersed IMC phase contained significantly higher Co and Cu contents and much lower Cr content, compared to the interfacial phase. When the reaction time was extended to 6 h (Figure 7b), a slight thickening of the interfacial (Fe,Cr,Co)Sn_2_ was observed, accompanied by a more pronounced spallation of (Fe,Co,Cu)Sn_2_ into the solder matrix.

After 12 h of reaction, as shown in Figure 7c, a similar interfacial microstructure was observed. According to the EPMA results (Table S3), the dispersed IMC phase of (Fe,Co,Cu)Sn_2_ contained lower Cr content but higher Co and Cu contents, compared to the interfacial (Fe,Cr,Co)Sn_2_ layer. The Cu present in the dispersed (Fe,Co,Cu)Sn_2_ phase originated from the SAC305 solder, indicating that Cu atoms substituted into Fe lattice sites within the FeSn_2_ structure. This substitution likely enhanced the phase stability of the IMC. Compared to the large dispersed (Fe,Cr,Co)Sn_2_ phase observed in the Sn/FeCoNiCr reaction (Figure 1d), the dispersed (Fe,Co,Cu)Sn_2_ phase formed in SAC305 exhibited a markedly finer microstructure. This observation suggests that the Cu addition promotes the nucleation of finer IMC particles, possibly by lowering the interfacial energy and stabilizing smaller grains. In contrast, Cr addition appears to favor the formation of larger IMC particulates, likely due to its repulsive interaction with molten Sn. Such repulsion suppresses the formation of smaller grains and facilitates the coarsening or growth of larger ones.

**Figure 8:**
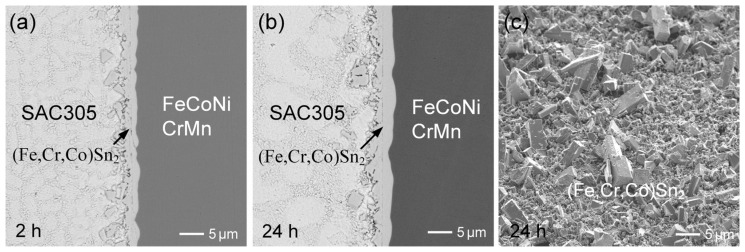
BEI micrographs of interfacial microstructures in SAC305/FeCoNiCrMn reactions at 250 °C for (**a**) 2 h and (**b**) 24 h. (**c**) The morphology of FeSn_2_ after aging for 24 h.

For comparison, Figure 8a,b show the interfacial microstructures in SAC305/FeCoNiCrMn reaction at 250 °C after aging for 2 h and 24 h, respectively. As reported in the prior study [30], the interface remained highly stable, with no significant microstructural evolution, and only a few (Fe,Cr,Co)Sn_2_ grains were observed dispersed in the solder near the interface. Similar to the reactions with Sn, the FeCoNiCrMn HEA exhibited superior interfacial stability compared to the FeCoNiCr MEA. Furthermore, the morphology of the (Fe,Cr,Co)Sn_2_ grains is shown in Figure 8c, consisting of a mixture of large faceted grains and small ones. In comparison to the Sn/FeCoNiCrMn system, the reduced grain size of (Fe,Cr,Co)Sn_2_ in the SAC305 solder is likely attributed to the presence of Cu, which may promote IMC nucleation and inhibit grain coarsening.

### 3.4. SAC305/FeCoNiMn Reactions

**Figure 9:**
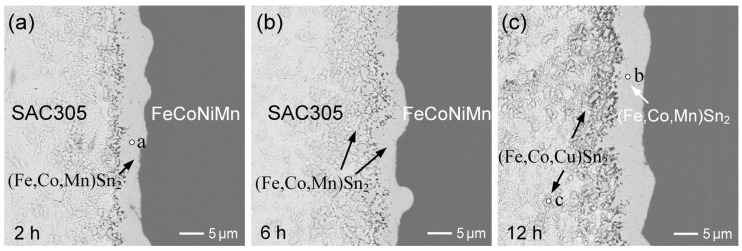
BEI micrographs of interfacial microstructures in SAC305/FeCoNiMn reactions at 250 °C for (**a**) 2 h, (**b**) 6 h, and (**c**) 12 h. The points labeled a–c represent the analyzed positions for EPMA, with the results summarized in Table S4.

The FeCoNiMn MEA was also reacted with SAC305 solder at 250 °C. Figure 9a–c show the interfacial results after aging for 2 h, 6 h, and 12 h, respectively. As the reaction progressed, the IMC spalling became increasingly significant. Notably, compared to the FeCoNiCr reaction, the spalled IMC grains were somewhat smaller in size. As listed in Table S4, the interfacial IMC layer was also identified as (Fe,Co,Mn)Sn_2_, containing a low Cu content of approximately 1.5 at.%. In contrast, the dispersed IMC phase was labeled as (Fe,Co,Cu)Sn_2_, with a higher Cu content of around 4 at.%, and negligible Mn content, likely due to the strong solubility of Mn in the solder matrix. These results are consistent with the reactions involving pure Sn (Figure 4), indicating that Mn in the alloys tends to promote IMC spalling behavior and grain size reduction. In addition, a chunky dispersed (Co,Ni)Sn_3_ phase was also observed in the SAC305 reactions with FeCoNiCr, FeCoNiMn, and FeCoNiCrMn, attributed to the significant dissolution of Co and Ni into the solder.

### 3.5. Contact Angle Analysis of SAC305 Solder on MEA/HEA Substrates

**Figure 10:**
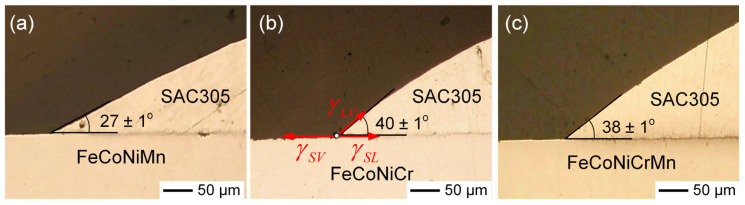
Cross-sectional images showing the contact angles of SAC305 solder on different MEA/HEA substrates after reflow at 250 °C for 20 s in an argon atmosphere: (**a**) FeCoNiMn, (**b**) FeCoNiCr, and (**c**) FeCoNiCrMn. Figure (**b**) also illustrates the corresponding surface energy relationships derived from Young’s equation.

The wettability between SAC305 solder and MEA/HEA substrates was evaluated through contact angle measurements, as shown in Figure 10a–c. Among the three substrates, the FeCoNiMn substrate exhibited the lowest contact angle (27 ± 1°), indicating superior wettability, while FeCoNiCr showed the highest (40 ± 1°). The contact angle for FeCoNiCrMn (38 ± 1°) was slightly lower than that of FeCoNiCr, which may be attributed to the reduced Cr content or the beneficial influence of Mn addition on interfacial wettability. This difference in wettability can be explained by Young’s equation:

(1) $$\gamma_{SV}=\gamma_{SL}+\gamma_{LV}\cos\theta$$

Here, $\gamma_{SV}$, $\gamma_{SL}$, and $\gamma_{LV}$ refer to the solid–vapor, solid–liquid, and liquid–vapor surface energies, respectively, and *θ* is the contact angle, as illustrated in Figure 10b. Since the liquid–vapor surface energy ($\gamma_{LV}$) of SAC305 is constant in all cases, variations in contact angle primarily reflect changes in solid–liquid interfacial energy ($\gamma_{SL}$). A lower contact angle corresponds to a lower solid–liquid interfacial energy, indicating stronger interfacial affinity interactions and improved adhesion between the solder and substrate.

The better wettability observed for FeCoNiMn is consistent with thermodynamic data. Mn exhibits a higher affinity toward Sn, which reduces the solid–liquid interfacial energy ($\gamma_{SL}$), enhances adhesion between the solder and substrate, and promotes the dissolution of the FeCoNiMn substrate into the solder. Conversely, Cr tends to form more stable native oxides and shows weaker affinity with Sn, leading to higher interfacial energy and poorer wettability, as evidenced by the higher contact angle on the FeCoNiCr substrate. These findings suggest that Mn addition improves interfacial adhesion and promotes more thermodynamically favorable solder spreading behavior, which may contribute to enhanced solder joint integrity.

## 4. Conclusions

The interfacial reactions of FeCoNiCr and FeCoNiMn MEAs with pure Sn and SAC305 solders were examined at 250 °C to investigate microstructure evolution during aging. For comparison, the FeCoNiCrMn HEA was also studied. Among the three systems, the Sn/FeCoNiCrMn reaction exhibited the most stable interface, maintaining a continuous (Fe,Cr,Co)Sn_2_ layer without IMC spallation. In contrast, the Sn/FeCoNiCr and Sn/FeCoNiMn systems showed significant IMC spalling into the solder matrix. In the Sn/FeCoNiCr system, the spalled (Fe,Cr,Co)Sn_2_ phase appeared as large, chunky particles, likely due to the repulsive interaction between Cr and molten Sn. Additionally, the substantial dissolution of Co and Ni from the substrate promoted the formation of large, plate-like (Co,Ni)Sn_3_ phases. In the FeCoNiMn system, the interfacial microstructure was more refined and stable. The presence of Mn promoted the dissolution of substrate elements into the solder and facilitated the formation of finer (Fe,Co,Mn)Sn_2_ particles, thereby reducing IMC grain size. Furthermore, in reactions with SAC305 solder, the formation of finer, dispersed (Fe,Co,Cu)Sn_2_ particles, compared to reactions with pure Sn, was observed. This was attributed to the Cu doping, which stabilized the IMC phase and enhanced nucleation, leading to smaller particle sizes.

Contact angle measurements with SAC305 solder revealed superior wettability on the FeCoNiMn substrate, due to the high Sn–Mn affinity, which lowers the interfacial energy and strengthens interfacial bonding. In contrast, the FeCoNiCr substrate exhibited a higher contact angle, reflecting weaker Sn–Cr interaction and poorer wetting. These results underscore the importance of elemental selection in designing MEA/HEA substrates to optimize interfacial reactions. By understanding the roles of elements such as Fe, Co, Ni, Mn, Cu, and Cr, it is possible to tailor interfacial microstructures and enhance the reliability and performance of solder joints.

## Data Availability

The original contributions presented in this study are included in the article/supplementary material. Further inquiries can be directed to the corresponding author.

## Supplementary Materials

The following supporting information can be downloaded at: https://www.mdpi.com/article/10.3390/ma18102379/s1, Tables S1–S4 show the EPMA compositional analysis for the reactions studied in this work. The analyzed positions are indicated in Figure 1, Figure 4, Figure 7 and Figure 9, respectively.
