# Supplementary material for: Liquid-State Interfacial Reactions of Lead-Free Solders with FeCoNiCr and FeCoNiMn Medium-Entropy Alloys at 250 °C
Source: Materials (Basel). 2025 May 20;18(10):2379. doi: 10.3390/ma18102379 (PMC12112771; doi:10.3390/ma18102379)
Supplement: Supplementary file 1 [file materials-18-02379-s001.zip › materials-3614471-supplementary.pdf]

materials

Supplementary material

Liquid-State Interfacial Reactions of Lead-Free Solders with Fe-CoNiCr and FeCoNiMn Medium-Entropy Alloys at 250 ºC

Chao-Hong Wang * and Yue-Han Li

Department of Chemical Engineering, National Chung Cheng University, Chiayi 621301, Taiwan
* Correspondence: chmchw@ccu.edu.tw

Academic Editor: Tomasz Czujko

Received: 14 April 2025
Revised: 16 May 2025
Accepted: 18 May 2025
Published: 20 May 2025

Citation: Wang, C.-H.; Li, Y.-H. Liquid-State Interfacial Reactions of Lead-Free Solders with FeCoNiCr and FeCoNiMn Medium-Entropy Alloys at 250 °C. *Materials* **2025**, *18*, 2379. https://doi.org/10.3390/ma18102379

Copyright: © 2025 by the authors. Licensee MDPI, Basel, Switzerland. This article is an open access article distributed under the terms and conditions of the Creative Commons Attribution (CC BY) license(https://creativecommons.org/licenses/by/4.0/).

Table S1. EPMA compositional analysis in the Sn/FeCoNiCr reactions at 250°C. The analyzed positions were indicated in Figure 1. The compositions of all these elements are presented in atomic percent (at.%).

Position	Fe (at%)	Co	Ni	Cr	Sn	phases
a	16.55	6.26	1.08	9.21	66.90	(Fe,Cr,Co)Sn_2
b	12.68	5.63	1.00	13.53	67.16	(Fe,Cr,Co)Sn_2
c	15.64	4.88	1.61	12.29	65.58	(Fe,Cr,Co)Sn_2
d	11.35	5.37	1.07	14.22	67.99	(Fe,Cr,Co)Sn_2
e	1.17	11.68	11.94	0.05	75.16	(Co,Ni)Sn_3

Table S2. EPMA compositional analysis in the Sn/FeCoNiMn reactions at 250°C. The analyzed positions were indicated in Figure 4. The compositions of all these elements are presented in atomic percent (at.%).

Position	Fe (at%)	Co	Ni	Mn	Sn	phases
a	18.45	9.30	2.88	3.63	65.74	(Fe,Co,Mn)Sn_2
b	19.10	8.74	1.09	5.26	65.81	(Fe,Co,Mn)Sn_2
c	1.33	14.10	8.76	0.19	75.62	(Co,Ni)Sn_3
d	1.07	13.52	10.31	0.24	74.86	(Co,Ni)Sn_3
e	21.56	6.46	1.96	5.15	64.87	(Fe,Co,Mn)Sn_2
f	16.20	7.19	0.84	8.02	67.75	(Fe,Co,Mn)Sn_2

Table S3. EPMA compositional analysis in the SAC305/FeCoNiCr reactions at 250°C. The analyzed positions were indicated in Figure 7. The compositions of all these elements are presented in atomic percent (at.%).

Position	Fe (at%)	Co	Ni	Cr	Cu	Sn	phases
a	17.38	3.23	1.60	10.17	1.66	65.96	(Fe,Cr,Co)Sn_2
b	18.23	7.31	1.78	1.94	4.21	66.53	(Fe,Co,Cu)Sn_2
c	18.40	5.84	1.62	8.75	1.23	64.16	(Fe,Cr,Co)Sn_2
d	20.87	5.34	0.58	2.59	3.88	66.74	(Fe,Co,Cu)Sn_2

Table S4. EPMA compositional analysis in the SAC305/FeCoNiMn reactions at 250°C. The analyzed positions were indicated in Figure 9. The compositions of all these elements are presented in atomic percent (at.%).

Position	Fe (at%)	Co	Ni	Mn	Cu	Sn	phases
a	13.65	8.92	2.83	3.79	1.50	69.31	(Fe,Co,Mn)Sn_2
b	21.75	8.20	1.48	2.08	1.24	65.25	(Fe,Co,Mn)Sn_2
c	19.02	9.57	1.26	0.13	3.88	66.14	(Fe,Co,Cu)Sn_2
